# Antifungal Effect of Probiotic *Lactobacillus casei* on Drug-Resistant Oral *Candida albicans* Isolated from Patients with Hematological Malignancy: an *in vitro* Study

**DOI:** 10.30476/dentjods.2022.94792.1812

**Published:** 2023-12-01

**Authors:** Seyedeh Saba Sharifzadeh, Ensieh Lotfali, Simin Lesan, Taraneh Farrokhnia

**Affiliations:** 1 Postgraduate Student, Dept. of Oral and Maxillofacial Medicine, Faculty of Dentistry, Islamic Azad University, Tehran Medical Branch, Tehran, Iran; 2 Dept. of Medical Parasitology and Mycology, School of Medicine, Shahid Beheshti University of Medical Sciences, Tehran, Iran; 3 Dept. of Oral Medicine, Faculty of Dentistry, Tehran Medical Sciences, Islamic Azad University, Tehran, Iran

**Keywords:** *Candida albicans*, Cell co-aggregation, Drug resistance, Hematological neoplasms, Probiotics

## Abstract

**Statement of the Problem:**

*Candida albicans* (*C. albicans*) is recognized as the most common opportunistic pathogen in patients with an impaired immune system, and due to the frequent use of antifungal medicine, a variety of drug-resistant species are developing. Probiotics are a part of the human microbiome and natural competitors of Candida by producing lactic acid, low pH, and other secreted metabolites. The role of probiotics in preventing fungal infections has always been discussed.

**Purpose:**

This study aimed to investigate the antifungal effect of *Lactobacillus casei* (*L. casei*) on fluconazole- and amphotericin B-resistant *C. albicans* species isolated from the oral cavity of acute myeloid leukemia patients.

**Materials and Method:**

In this experimental study, eight strains of fluconazole- and amphotericin B-resistant *C. albicans* were used. The antifungal effects of probiotic *L. casei* and nystatin were measured by the co-aggregation method 1, 2, and 4 h after beginning the study. After each hour of exposure, *C. albicans* and *L. casei* colonies were counted.

**Results:**

*L. casei* had a significant ability to aggregate with both fluconazole- and amphotericin B-resistant *C. albicans* in all designated intervals, which increased with time. In the first hour of the study, no significant difference was observed between the effects of *L. casei* on the two drug-resistant strains. However, as time passed, it had a more significant antifungal effect on fluconazole, compared to amphotericin B resistant species (*p* Value<0.001). Cell counts showed that the number of fungal cells decreased significantly as time passed (*p*< 0.001).

**Conclusion:**

*L. casei* had a significant ability to aggregate with both drug-resistant *C. albicans* species and showed higher antifungal activity on fluconazole-resistant than amphotericin B-resistant species.

## Introduction

*Candida albicans* (*C. albicans*) is recognized as the most common opportunistic pathogen in impaired immune system patients, and its prevalence is increasing with the growing number of immunocompromised people [ 1 - 2 ]. It is clear that patients with a weakened immune system, due to chemotherapy and radiotherapy for cancer treatment, are highly prone to oral candidiasis. The quality of these patients’ life is completely affected by fungal infections, and they may experience severe, invasive, and even life-threatening side effects following the entry of Candida species into their bloodstream (candidemia) [ 1 - 4 ]. The colonization of *C. albicans* in immune-deficient patients, especially in hematological malignancies, can affect the final treatment results [ 5 ]. Currently, following the frequent, long-term, and prophylactic use of antifungal medications in patients with candidiasis, the variety of drug-resistant Candida strains is increasing. Therefore, finding an alternative way to control and prevent this group of fungal infections is suggested [ 4 , 6 ]. Some studies [ 7 - 10 ] show that Candida species colonization is reduced following the consumption of foods containing probiotics [ 7 ]. Lactobacillus species are among probiotics that inhibit the formation and growth of Candida biofilm and reduce the symptoms of infection. The role of probiotics in preventing infectivity is not fully understood, and it is currently hypothesized that probiotics inhibit the growth of fungal cells by competing for food, occupying the sites required for Candida attachment, and producing antimicrobial agents. Probiotics can also improve the mucosal immune system [ 8 - 10 ]. Therefore, this study aims to investigate the role of probiotic *Lactobacillus casei* (*L. casei*) in the prevention of fungal lesions in the form of co-aggregation. 

## Materials and Method

### *L. casei* and culture conditions

In this *in vitro* experimental study, the probiotic *L. casei* (PTCC number 1608) was used, provided by the Iranian Research Organization for Science and Technology. The *L. casei* was cultured under anaerobic conditions in Man Rogosaf Sharpe (MRS, SIGMA, USA) medium at 37˚C for 24 h. The isolated *L. casei* colonies were transferred to the MRS medium (5 ml) and incubated at 37˚C for 48 h. After the incubation, *L. casei* was stocked at 20˚C in glycerol. 1 ml of *L. casei* was relocated to 5 ml of MRS broth medium for recultivation. Finally, L-cysteine (50 μl) ​​was added. The microtubes were located in a shaker incubator (16B; KTG laboratory equipment) at 37˚C for 20 h [ 8 ].

### *C. albicans* and culture conditions

A microbiologist isolated the *C. albicans* from the oral cavity of patients with acute myeloid leukemia admitted to the Taleghani Hospital of Shahid Beheshti University (Tehran, Iran). These patients did not show any improvement in oral lesions following the administration of fluconazole and amphotericin B, either clinically or microscopically (in terms of colony count, cell numbers, and fungal wall destruction). Sampling was done with two swabs prepared from the oral lesions caused by Candida species. They were placed in 1 cc of distilled water and transferred to the laboratory. A direct slide was prepared from one swab and the second swab was prepared on Saburo Dextrose Agar (SDA, SIGMA, US-A) medium. Minimum inhibitory concentration results were determined according to the CLSI-M27-S3 (Clinical & Laboratory Standards Institute) and showed that the strains were resistant to fluconazole and amphotericin B (13). Candida species were stored at ‒70˚C in Tryptic Soy Broth (SIGMA, USA). To start the study, Candida species were cultured in SDA and transferred to Saburo Dextrose Broth (SDB) for 24 h at 37˚C. Eight strains of fluconazole- and amphotericin B-resistant *C. albicans* were used, and the antifungal effects of *L. casei* (study groups) were measured, in comparison with nystatin (control groups), by the co-aggregation method 1, 2, and 4 h after the study began. The study groups included (1) *L. casei* and fluconazole-resistant *C. albicans*, (2) *L. casei* and amphotericin B resistant *C. albicans*, (3) nystatin and fluconazole-resistant *C. albicans*, and (4) nystatin and amphotericin B resistant *C. albicans* [ 8 ]. 

### Co-aggregation of *C. albicans* and *L. casei*

The co-aggregation was assessed by a spectrophotometer (PD-303; Apple model). At first, the mixture of *L. casei* and *C. albicans* was incubated for 1, 2, and 4 h. Then, the co-aggregation ratio was determined consistent with the methodology proposed by Salari *et al*. [ 8 ] Initially, the detached colonies of 24-h *L. casei* were moved to 5 ml MRS broth medium and were incubated in a shaker incubator (84 RPM; 24 h; 37˚C) in anaerobic carriage. The *C. albicans* was isolated from SDA and cultured in SDB at 37˚C for 24 h. Then, the microtubes containing *L. casei* and *C. albicans* were centrifuged (855 RPM; 10 min, 25˚C). The supernatant was removed and the sediment was washed three times in phosphate buffer saline (PBS, SIGMA, USA) and suspended in 10 mmol/L PBS (at pH=7). The optical absorbance was measured by a spectrophotometer at the specific wavelength of 600 (OD600 nm) nanometers equivalent to the McFarland standard (10^8^ CFU/mL for Lactobacilli and 10^6^ CFU/mL for Candida species). In total, 1ml of each *L. casei* and 1 ml of *C. albicans* were totally mixed and incubated in a shaker incubator (100 rpm; 37˚C; for 1, 2, and 4 h). Then, the optical density (OD) measurement was performed with spectrophotometer at OD600 nm. Each experiment was repeated three times [ 8 ].

The percentage of co-aggregation was calculated based on the following equation:

$\mathrm{Co}-\mathrm{aggregation\%:}[({\mathrm{OD}}_{0}-{\mathrm{OD}}_{h})/{\mathrm{OD}}_{0}]\times 100$

OD_0_: the absorption amount of the complex suspension of *L. casei* with *C. albicans* at the beginning of the trail (0 h).

OD_h_: the absorption amount of the complex solutions at various times

### *L. casei* and *C. albicans* cell counts

To evaluate the cell number of *C.albicans* and *L.casei* 1, 2, and 4h after the study began (after measuring the absorbance of the solution by the spectrophotometer OD60-0nm), some of the solution containing *L.casei* and *C.albicans* was removed with an inoculation needle and transferred to the culture medium. After 24h, the number of cells was determined by counting the colonies of each species separately and calculating the number of cells by calculating each colony in a certain coefficient of dilution (coefficient of dilution was prepared based on the diameter of the needle loop). Small colonies were belonged to *L.casei* and larger colonies revealed *C.albicans*.

### Data analysis

Data were analyzed by the SPSS 11 software through one-way ANOVA and Tukey HSD tests.

## Results

### Co-aggregation percentage between *L. casei* and two drug-resistant *C. albicans* in 1, 2, and 4 hours of study

The normality of data distribution was checked by Kolmogorov-Smirnov test. Based on the test results, the distribution of all data was normal. One-way ANOVA res-ults showed that in all three times (1, 2, and 4 h), *L. casei* had a significant ability to aggregate with drug-resistant *C. albicans*, which increased with time (*p*< 0.001). 

### First hour of the study

**Figure 1:**
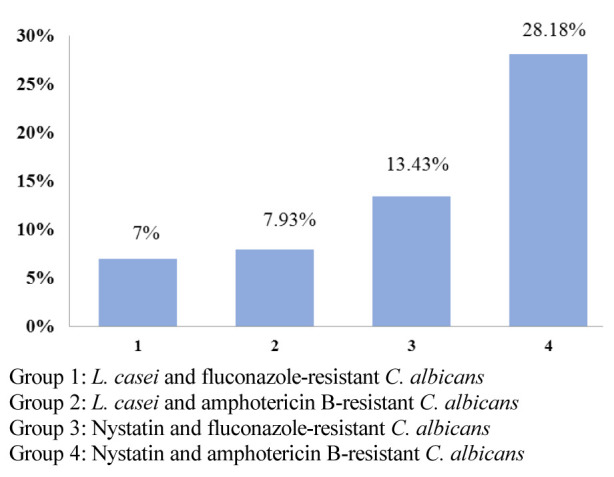
Co-aggregation percentage of *L. casei* with drug-resistant *C. albicans* species in comparison with Nystatin, in the first hour of the study

There was no significant difference between the co-aggregation degree of *L.casei* with fluconazole-resistant and amphotericin B-resistant *C. albicans*, and the antifungal effect was almost similar. At the same time, compared to nystatin, *L. casei* had a significantly lower antifungal effect on amphotericin B resistant species (*p*< 0.001). On the other hand, nystatin had a slightly more antifungal effect on fluconazole-resistant species when compared to *L. casei*, but the difference was not significant (Figure 1).

### Second hour of the study

**Figure 2:**
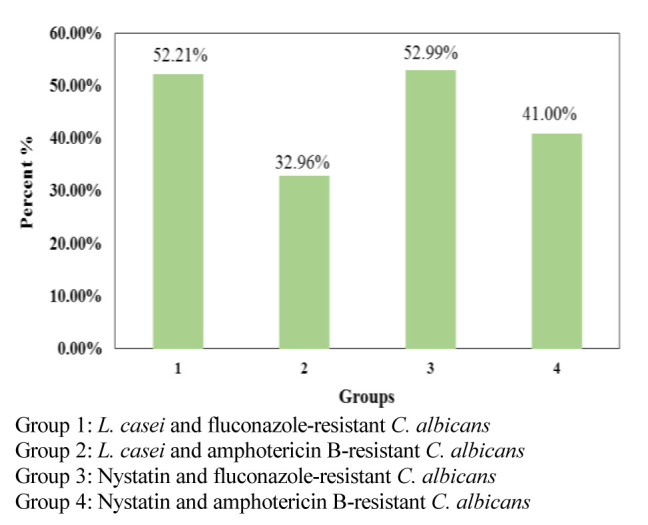
Co-aggregation percentage of *L. casei* with drug-resistant *C. albicans* species in comparison to Nystatin, in the second hour of the study

Herein, a significant difference was monitored between the co-aggregation percentage of *L. casei* with a fluconazole-resistant, compared to amphotericin B-resistant *C. albicans* (*p*< 0.001). Moreover, *L. casei* had greater co-aggregation ability with fluconazole-resistant species than amphotericin B-resistant strains. At this time, no significant difference was observed between the antifungal results of *L. casei* and nystatin on amphotericin B and fluconazole-resistant species. However, the findings revealed that in amphotericin B-resistant species, the effect of nystatin was slightly better, and in fluconazole-resistant species, *L. casei* had an antifungal effect similar to that of nystatin (Figure 2).

### Fourth hour of the study

**Figure 3:**
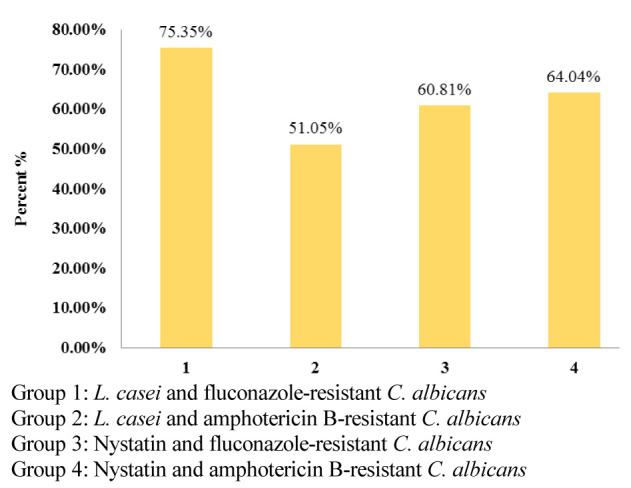
Co-aggregation percentage of *L. casei* with drug-resistant *C. albicans* species in comparison to Nystatin, in the fourth hour of the study

In the fourth hour, *L. casei* had a significantly higher co-aggregation rate with fluconazole-resistant than amphotericin B-resistant species (*p*< 0.001). Furthermore, compared to nystatin, *L. casei* had a significantly greater antifungal effect on fluconazole-resistant species (*p*< 0.001). However, this comparison was completely reversed in the *L. casei* and nystatin groups on amphotericin B-resistant *C. albicans*, leading to a better effect of nystatin than *L. casei* (*p*< 0.001). At this stage, the co-aggregation of *L. casei* with fluconazole-resistant strains was better than all groups and had a significant antifungal effect, in comparison with other groups of the study (*p*< 0.001) (Figure 3).

### Cell counting of Lactobacilli casei and drug-resistant *C. albicans* after 1, 2, and 4 hours of exposure

**Figure 4:**
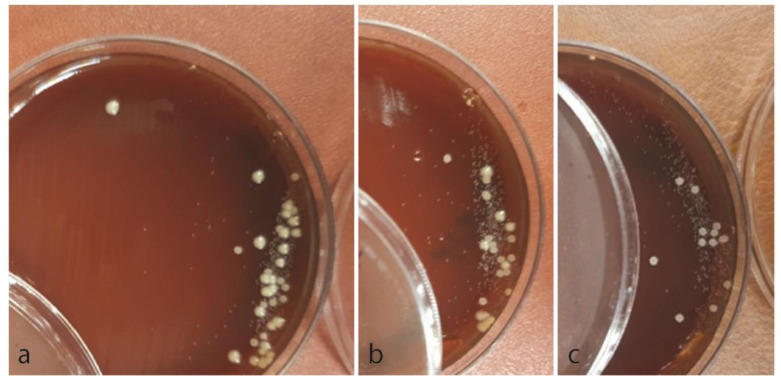
**a:** Smaller colonies were belonged to *L. casei* and larger colonies revealed *C. albicans*. control group, **b:** *L. casei* and amphotericin B resistant *C. albicans*, **c:** *L. casei* and fluconazole resistant *C. albicans*

Cell number of *L. casei* and resistant *C. albicans* after 1, 2, and 4 hours of exposure showed that the amount of *L. casei* and drug-resistant *C. albicans* cells decreased significantly over time (*p*< 0.001). It was also found that the amount of fluconazole-resistant *C. albicans* and *L. casei* cells decreased more, in comparison with *L. casei* and amphotericin B-resistant cells in the fourth hour of the study (*p*< 0.001) (Figure 4).

## Discussion

Due to the increasing prevalence of oral candidiasis in immunodeficient patients and the development of drug-resistant Candida species, the use of prophylactic components, such as probiotics, for preventing fungal infections has always been proposed [ 11 - 14 ]. The role of probiotics in this field is generally recognized, and various studies have revealed its role in reducing the signs and symptoms of oral candidiasis [ 15 - 16 ]. Several experiences have revealed antifungal effects of different probiotics on oral *C. albicans* [ 8 - 9 , 17 - 18 ]. This study tested the antifungal effect of probiotic *L. casei* on fluconazole- and amphotericin B-resistant *C. albicans* by evaluating the degree of co-aggregation and the colony counting method. In the study of Salari *et al*. [ 8 ], the ability of co-aggregation was seen between *L. acidophilus* and *L. plantarum* with all tested Candida strains, and their co-aggregation percentage increased over time. The highest co-aggregation rate was observed between *C. krusei* and two probiotics, which was lower than that of *C. albicans*. In a study by Jørgensen *et al*. [ 19 ], *L. reuteri* with *C. tropicalis* and *C. krusei* had the greatest co-aggregation ratio. Chew *et al*. [ 20 ] also stated that *L. reuteri* showed a mainly greater co-aggregation ratio versus *C. glabrata* species, in comparison with *L. rhamnosus*. Therefore, it appears that co-aggregation levels are exclusive for each species of Lactobacilli [ 8 ]. In the next part of this study, colony counting was performed 1, 2, and 4 hours after the study began. Based on counting drug resistant *C. albicans* colonies, it was found that the number of cells reduced significantly over time, and the number of fluconazole-resistant colonies decreased more significantly, in comparison with amphotericin B-resistant colonies in the fourth hour of the study (*p*< 0.001). It was consistent with the co-aggregation results, which showed that in the fourth hour, *L. casei* had greater co-aggregation ability with fluconazole-resistant strains than amphotericin B-resistant strains. The findings of a study by Srivastava *et al*. [ 21 ], which tested the inhibitory effect of *L. plantarum* on *C. albicans* and *Streptococcus mutans* (*S. mutans*) by counting the colonies, showed that *L. plantarum* significantly stopped the formation of *C. albicans* biofilm alone and in combination with *S. mutans*. Probiotics inhibit *C. albicans* activity in different ways, including the inhibition of fungal adhesion to surfaces, production of acids, bacteriocins, biosurfactants, as well as hydrogen peroxide, and co-aggregation. Co-aggregation is one of the hallmarks of primary biofilm formation since it involves an adhesion-receptor interaction between microbial cell surfaces. In fact, the reduction in cell-dependent adhesion is probably due to the simultaneous co-aggregation of *L. casei* and *C. albicans* species. Interestingly, good initial adhesion of certain strains of probiotics, such as *L. gasseri*, *L. cryspatus*, or *L. vaginalis*, is not compatible with a good inhibition of *C. albicans* adhesion. It indicates that *C. albicans* adhesion is minimized due to the changes in the surface of epithelial cells or the effect of probiotics on the adhesion ability of the pathogen [ 16 , 22 ]. Previous study has also shown that *L. casei* has the ability to produce Interleukin-12, which stimulates natural killer cells (first line of defense) to respond against the infection and their proper function plays an important role in the prognosis of the disease [ 18 ]. In a study by Villena *et al* [ 9 ], *L. casei* was found to prevent the damage caused by inflammatory responses by producing interleukin-10 (IL-10). The IL-10 is a potent anti-inflammatory regulatory cytokine that has a beneficial effect on the host’s response against fungal infections. In the early stages of infection, cytokines can interfere with the antifungal function of phagocytes and the production of other proinflammatory cytokines. However, in the later stages of infection, the increase in IL-10 production may help relieve the inflammatory response. Therefore, the use of this probiotic as a supplement with the mechanism of increasing IL-10 prevents the damage caused by inflammatory responses [ 9 ]. The difficulty of Lactobacillus bacteria growth in culture medium and the sensitivity of probiotics to environmental conditions were some of the possible limitations. A novel feature of our survey was the comparison between the antifungal effects of *L. casei* on fluconazole- and amphotericin B-resistant *C. albicans*. These species were isolated from the oral cavity of AML patients. The incidence of oral candidiasis is associated immunity reduction followed by a chemotherapy regimen in patients with hematological malignancies.

## Conclusion

The findings revealed that *L. casei* had a significant ability to co-aggregate with both drug-resistant *C. albicans* species and showed higher antifungal activity on fluconazole-resistant strains than amphotericin B-resistant species. Further investigations are recommended for assessing the antifungal properties of other Lactobacillus species on drug-resistant *C. albicans* and recognizing the precise mechanisms of their action, as well as performing antifungal susceptibility patterns in models of infected animal. 
